# Prognostic and clinicopathological significance of SNHG20 in human cancers: a meta-analysis

**DOI:** 10.1186/s12935-020-01403-8

**Published:** 2020-07-11

**Authors:** Hanlong Zhu, Si Zhao, Ruonan Jiao, Huishan Wang, Ruiyi Tang, Xiaochao Wu, Fei Wang, Xianxiu Ge, Quanpeng Li, Lin Miao

**Affiliations:** Medical Centre for Digestive Diseases, The Second Affiliated Hospital of Nanjing Medical University, Nanjing, 210011 Jiangsu People’s Republic of China

**Keywords:** SNHG20, lncRNA, Cancer, Prognosis, Meta-analysis

## Abstract

**Background:**

It has been widely reported that the expression levels of SNHG20 are elevated in diverse types of cancers, indicating that SNHG20 may participate in cancer initiation and development. Besides, accumulating evidence reveals that SNHG20 overexpression is also connected with poor clinical outcomes among cancer patients. Herein, we carry out a systematic meta-analysis to further determine the prognostic and clinical significance of SNHG20 expression in various human cancers.

**Methods:**

Qualifying publications were selected by searching for keywords in PubMed, Embase, Web of Science and Cochrane Library databases, up to September 1, 2019. Pooled hazard ratio (HR) or odds ratio (OR) with corresponding 95% confidence interval (CI) was computed to estimate the strength of association between SNHG20 and survival of cancer patients or clinicopathology using Stata 14.0 software.

**Results:**

In total, 15 studies encompassing 1187 patients met the inclusion criteria were ultimately enrolled for analysis. According to the meta-analysis, patients with high SNHG20 expression were markedly linked to poorer overall survival (OS) (pooled HR = 2.47, 95% CI 2.05–2.98, P = 0.000) and disease-free survival/recurrence-free survival/progression-free survival (DFS/RFS/PFS) (pooled HR = 2.37, 95% CI 1.60–3.51, P = 0.000). Additionally, regarding clinicopathology of patients, enhanced SNHG20 was correlated with advanced tumour‐node‐metastasis (TNM) stage (OR = 2.80, 95% CI 2.00–3.93, P = 0.000), larger tumor size (OR = 3.08, 95% CI 2.11–4.51, P = 0.000), positive lymph nodes metastasis (OR = 2.99, 95% CI 2.08–4.31, P = 0.000), higher tumor stage (OR = 4.51, 95% CI 2.17–9.37, P = 0.000) and worse histological grade (OR = 1.95, 95% CI 1.44–2.63, P = 0.000), but not with gender, smoking status or distant metastasis.

**Conclusions:**

Up-regulated SNHG20 expression is ubiquitous in different kinds of cancers. Moreover, up-regulated SNHG20 expression is capable of serving as an innovative predictive factor of inferior clinical outcomes in cancer patients. Nevertheless, higher-quality multicenter studies are required to corroborate our results.

## Background

Cancer has become one of the common chronic diseases that seriously threatens human health and imposes an immense burden on society. The upward trend of cancer gives rise to worldwide concern, with almost 1,762,450 newly diagnosed cancer cases and approximately 606,880 cancer-related deaths in the United States in 2019 [1]. Although the survival benefit of multidisciplinary synthetic therapy is recognized, the prognosis in patients with terminal stages of cancer remains unsatisfactory [2]. To this end, the identification of an accurate biomarker for cancer prognosis is of great clinical value, which can be applied to early diagnosis and targeted therapy for clinical practice.

More recently, with the advent of next-generation sequencing technologies, long noncoding RNAs (lncRNAs), as a new category of noncoding transcripts, have come into the spotlight [3]. By definition, lncRNAs constitute a large and heterogeneous subset of RNAs that are distinguished by a length of greater than 200 nucleotides and the absence of protein-coding capability [4]. They were once regarded as simply genomic “junk” so that they having been underappreciated for a long time [5]. Nonetheless, lncRNAs have emerged as functional molecules, which serve pivotal roles in diverse biological processes, with clear relevance to cancer [5]. Additionally, accumulating shreds of evidence unveil that lncRNAs can exert oncogenic or tumor-suppressing effects in tumorigenesis and progression [6], suggesting that lncRNAs may be candidate markers for cancer diagnosis, prognosis, and therapeutics.

The small nucleolar RNA host gene 20 (SNHG20) has arrested our attention among the lncRNAs, which stems from chromosome 17q25.2 and harbors 2183 basepairs [7]. Initially, SNHG20 was discovered in hepatocellular carcinoma (HCC) and has been proved to function as an oncogene in HCC [8]. Subsequently, a growing body of research has identified that aberrant SNHG20 expression definitely interacted with prognostic outcomes and clinicopathological characteristics in patients with many kinds of malignancies, including bladder cancer [9], osteosarcoma [10], glioma [11], colorectal cancer [12], gastric cancer [13], lung cancer [14], cervical cancer [15], esophageal carcinoma [16], oral carcinoma [17], nasopharyngeal carcinoma [18], ovarian cancer [19], and laryngeal carcinoma [20]. Consistently, overexpression of SNHG20 could promote cell-cycle, proliferation, invasion, and migration of tumor cells via different mechanisms, while up-regulated SNHG20 is an unfavorable prognostic factor [7]. It should be taken into account that most individual studies are restricted by controversial and discrete conclusions as well as small sample size. For the sake of comprehensively validating the underlying prognostic and clinicopathological role of SNHG20 in various malignancy patients, a quantitative meta-analysis is therefore undertaken.

## Materials and methods

### Literature search strategies

Up to September 1, 2019, potential eligible literature were systematically retrieved in four authoritative databases including PubMed, Embase, Web of Science and Cochrane Library databases to obtain pertinent articles regarding prognostic and clinicopathological features of SNHG20 among various tumors. The searched keywords in variably combinations were as following: (“small nucleolar RNA host gene 20” OR “SNHG20”) AND (“cancer” OR “carcinoma” OR “tumor” OR “neoplasm”) AND (“prognosis” OR “prognostic”). The reference lists of included studies were also checked to identify potential relevant papers.

### Inclusion and exclusion criteria

The research involved in this meta-analysis were asked to meet the following preassigned criteria: (1) investigated the roles of SNHG20 in multiple human tumors, (2) detected the expression levels of SNHG20 in cancer tissue, (3) divided the patients into dichotomous groups according to the specific criteria for SNHG20 expression levels, (4) reported data related with the clinicopathological characteristics and prognostic information of the patients, and (5) had sufficient data for calculating the hazard ratios (HR) with corresponding 95% confidence intervals (CI). All these studies were not included because of the any of the following reasons: (1) stated reduplicative research, (2) offered insufficient or unavailable data, (3) were reviews, letters, case reports, editorials, expert opinions, conference reports, and animal experiments, and (4) published in a non-English language.

### Literature screening and data extraction

Two investigators (Hanlong Zhu, Si Zhao) independently screened the literature following the prespecified criteria described above and extracted the data. Any conflicts were resolved through consensus with a third scholar (Ruonan Jiao). The following information was collected from each enrolled study: lead author name, publication year, region, carcinoma type, sample size (high/low), SNHG20 assessment method and the cut-off approximations for SNHG20 expression levels, the clinicopathological parameters including age, gender, smoking status, tumour-node-metastasis (TNM) stage, tumor size, lymph node metastasis, tumor stage, histological grade, and distant metastasis, together with HR and 95% CI for overall survival (OS), disease-free survival (DFS), recurrence-free survival (RFS) and progression-free survival (PFS). If only Kaplan–Meier curves existed in some articles, HR and 95% CI were determined with available data using the published method [21, 22].

### Quality evaluation

The quality of eligible publications was calculated based on the Newcastle–Ottawa Scale (NOS) that evaluated the selection of cohorts, comparability as well as exposure or outcome and had a score ranging from 0 to 9. Studies with higher or equal to 6 points could be considered as high quality (Table 1).

**Table 1:** Characteristics of included studies in the meta-analysis

Author	Year	Study region	Recruitment time	Cancer type	Age (%)	No. (high/low)	Outcome	Detection method	Cut-off value	Source of HR	NOS score
Li et al.	2016	China	2006–2011	Colorectal cancer	≥ 65 (52.3%)	107 (54/53)	OS	qRT-PCR	NA	Data in paper	7
Li et al.	2017	China	2007–2011	Hepatocellular carcinoma	> 65 (25.0%)	96 (50/46)	OS	qRT-PCR	Median	Survival curves	8
Wang et al.	2018	China	2016–2017	Osteosarcoma	≥ 18 (88.2%)	32 (18/14)	OS	qRT-PCR	Median	Survival curves	8
Zhang1 et al.	2016	China	2006–2009	Hepatocellular carcinoma	> 55 (41.7%)	144 (98/46)	OS DFS	ISH	NA	Data in paper	6
Cui et al.	2018	China	2012–2014	Gastric cancer	> 55 (50.0%)	56 (28/28)	OS DFS	qRT-PCR	NA	Survival curves	6
Chen et al.	2017	China	2013–2015	Lung cancer	> 65 (52.4%)	42 (21/21)	OS PFS	qRT-PCR	Median	Survival curves	7
Guo et al.	2018	China	NA	Cervical cancer	≥ 45 (47.3%)	93 (47/46)	OS	qRT-PCR	NA	Survival curves	7
Zhang et al.	2018	China	NA	Esophageal carcinoma	≥ 60 (67.5%)	80 (37/43)	OS	qRT-PCR	Median	Survival curves	6
Gao et al.	2018	China	2008–2013	Oral carcinoma	> 60 (45.0%)	40 (20/20)	OS	qRT-PCR	NA	Data in paper	8
Gao et al.	2019	China	NA	Glioma	≥ 60 (30.8%)	78 (33/45)	OS	qRT-PCR	Mean	Survival curves	8
Sun et al.	2018	China	2011–2013	Nasopharyngeal carcinoma	> 50 (52.7%)	55 (28/27)	OS	qRT-PCR	Median	Survival curves	6
Wang et al.	2019	China	NA	Epithelial ovarian cancer	> 55 (43.3%)	60 (38/22)	OS	qRT-PCR	NA	Survival curves	6
Zhang2 et al.	2018	China	NA	Osteosarcoma	> 18 (61.4%)	140 (70/70)	OS	qRT-PCR	NA	Data in paper	7
Li1 et al.	2019	China	NA	Laryngeal carcinoma	≥ 60 (58.9%)	56 (28/28)	OS	qRT-PCR	NA	Survival curves	8
Li2 et al.	2019	China	2011–2017	Glioma	≥ 50 (52.8%)	108 (54/54)	OS RFS	qRT-PCR	Median	Data in paper	6

*NO* number, *HR* hazard ratio, *NOS* Newcastle–Ottawa Scale, *NA* not available, *OS* overall survival, *DFS* disease-free survival, *RFS* recurrence-free survival, *PFS* progression-free survival, *qRT*-*PCR* quantitative reverse transcription polymerase chain reaction, *ISH* in situ hybridization

### Statistical analysis

The pooled HR with corresponding 95% CI was utilized to estimate the relationship between SNHG20 expression and patients’ prognosis. While the effect of SNHG20 expression on clinicopathological features was described as the combined odds ratio (OR) and matching 95% CI. Cochran’s Q and I^2^ tests were applied for checking the heterogeneity of the results. A P value < 0.1 suggested having statistical significance, whereas I^2^ values > 50% indicated the existence of significant heterogeneity. When there was homogeneous data, the fixed-effect framework was adopted, otherwise, the random-effect model was employed. Besides, probable publication bias was quantified with conducting Begg’s test and Egger’s test, respectively. Sensitivity analysis was also done to investigate the stability of the accumulated results. All analyses were carried out using Stata 14.0 software. P value < 0.05 was regarded as being statistically significant.

## Results

### Data selection and characteristics

**Fig. 1:**
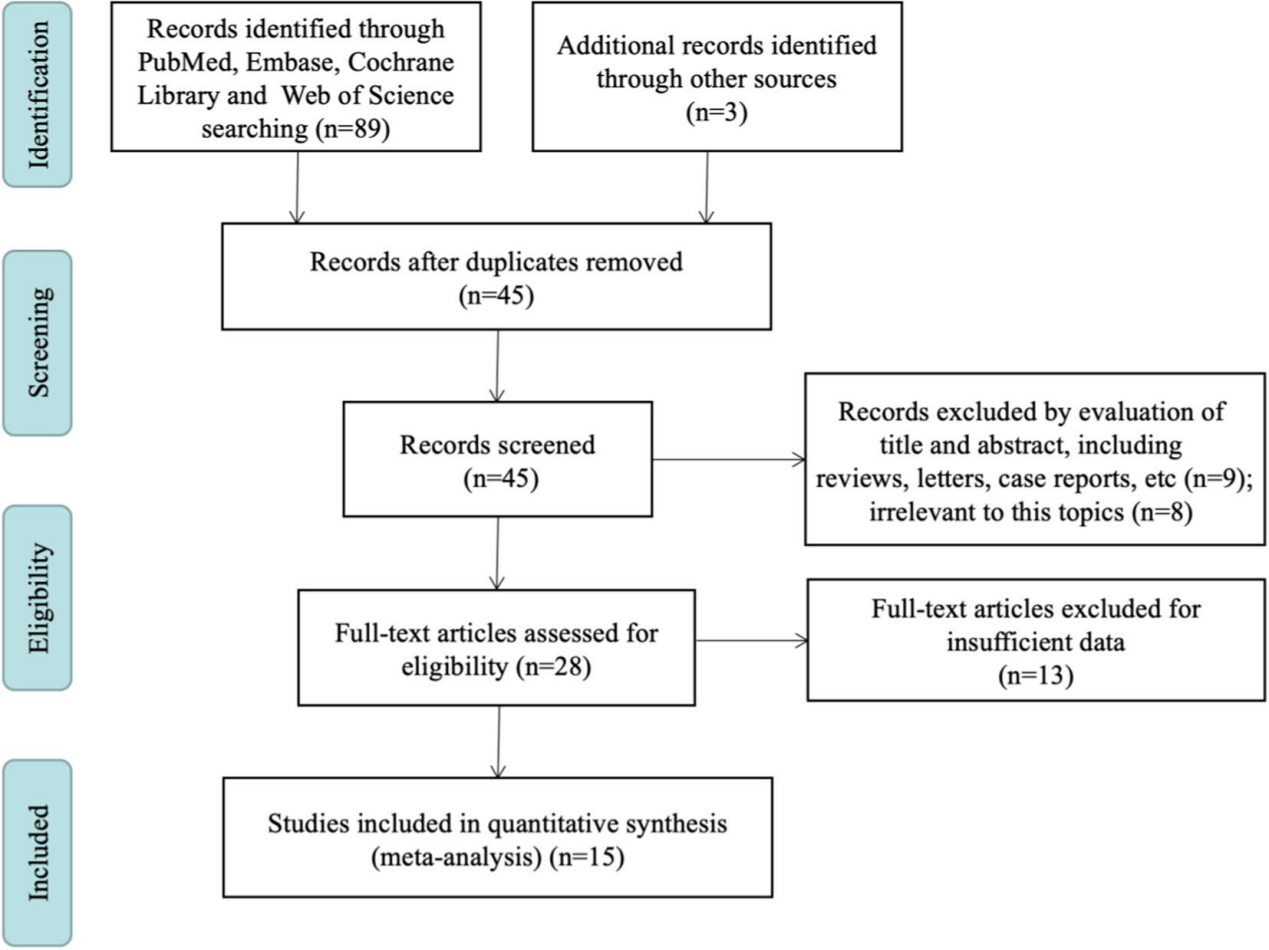
Flow diagram of the process for study selection

According to the search strategy, 89 relevant records were initially retrieved from four electronic databases. Three publications were enrolled by manually searching the reference lists. After ruling out the duplicates, 45 studies were left for further assessment. Next, 17 papers were directly removed by carefully scanning titles and abstracts. For the remaining 28 articles, 13 articles were excluded owing to lack of sufficient data. Ultimately, 15 studies showing agreement with the inclusion criteria were selected for entering in the meta-analysis (Fig. 1).

The attributes of the research studies involved in the present analysis are summarized in Table 1. These studies containing 1187 cancer patients had an accrual period between 2016 and 2019 and sample sizes varying from 32 to 144 (mean, 79). Each and every research study was performed in China; two studies referred to hepatocellular carcinoma [8, 23], two studies involved osteosarcoma [10, 24], two studies touched upon glioma [11, 25], and the remaining nine studies related to colorectal cancer [12], gastric cancer [13], lung cancer [14], cervical cancer [15], esophageal carcinoma [16], oral carcinoma [17], nasopharyngeal carcinoma [18], ovarian cancer [19], and laryngeal carcinoma [20]. The whole subjects registered were separated into high and low SNHG20 group on the basis of the SNHG20 measurement results. Moreover, 14 studies used quantitative reverse transcription polymerase chain reaction (qRT-PCR) for the detection of SNHG20 expression in tumor tissues while only one study chose in situ hybridization (ISH). Most articles preferred to exploit the median value as the Cut-off value for high or low SNHG20 expression. Regarding survival outcomes, all of the cohorts reported patients’ OS, of which four cohorts simultaneously presented DFS/RFS/PFS. Among the studies, HR and 95% CI were provided in five original articles and indirectly reckoned from survival curves in the rest of ten papers. Overall, all these qualified studies were recognized to be of high quality in this meta-analysis.

### Association between SNHG20 expression and survival of cancer patients

#### SNHG20 expression and OS

**Fig. 2:**
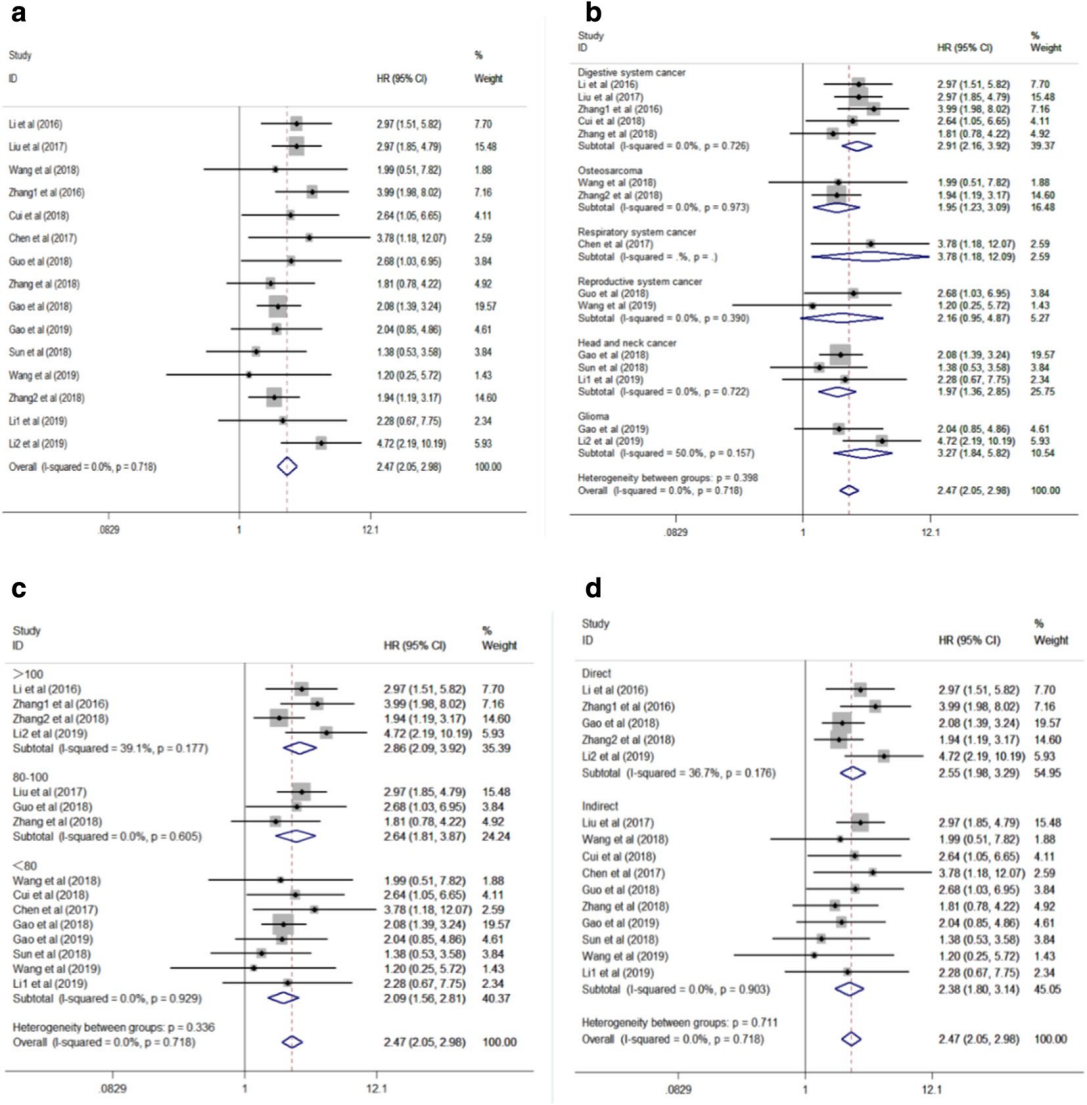
Forest plots for the association between SNHG20 expression and OS. **a** overall; **b** cancer type; **c** sample size; **d** extracted method. *OS* overall survival, *HR* hazard ratio, *95% CI* 95% confidence interval

A total of fifteen studies comprising 1187 patients focused on assessing the effect of SNHG20 overexpression on OS in various kinds of cancer. As illustrated in Fig. 2a, a fixed-effect framework was applied because of the lack of significant heterogeneity among these studies (I^2^ = 0.0%, P = 0.718). The pooled HR suggested that the high SNHG20 expression group showed a statistically obvious decline in OS (pooled HR = 2.47, 95% CI 2.05–2.98, P = 0.000). In addition, subgroup analyses were performed regarding cancer types, sample sizes and data extraction methods to further analyze the predictive value of SNHG20 (Fig. 2b–d, Table 2). In the stratified analysis by type of cancers, promoted SNHG20 expression status was closely related to worse OS of the patients with respiratory system cancers (pooled HR = 3.78, 95% CI 1.18–12.09, P = 0.025, fixed-effect), gliomas (pooled HR = 3.27, 95% CI 1.84–5.82, P = 0.000, fixed-effect), digestive system cancers (pooled HR = 2.91, 95% CI 2.16–3.92, P = 0.000, fixed-effect), head and neck cancers (pooled HR = 1.97, 95% CI 1.84–5.82, P = 0.000, fixed-effect) and osteosarcomas (pooled HR = 1.95, 95% CI 1.23–3.09, P = 0.005, fixed-effect), apart from reproductive system cancers (pooled HR = 2.16, 95% CI 0.95–4.87, P = 0.065, fixed-effect). When the studies were categorized according to sample sizes, a significant connection was observed between SNHG20 upregulation and inferior OS in large sample sizes (> 100, pooled HR = 2.86, 95% CI 2.09–3.92, P = 0.000, fixed-effect), middle sample sizes (80-100, pooled HR = 2.64, 95% CI 1.81–3.87, P = 0.000, fixed-effect) or small sample sizes(< 80, pooled HR = 2.09, 95% CI 1.56–2.81, P = 0.000, fixed-effect), demonstrating that larger sample sizes might devote to more robust and accurate results. As for different data extraction methods, the subgroup analysis unveiled that the prognostic value of SNHG20 on the OS was not influenced by data extraction methods, that is, the HR provided in the papers (pooled HR = 2.55, 95% CI 1.98–3.29, P = 0.000, fixed-effect) or extracted from the survival curves (pooled HR = 2.38, 95% CI 1.80–3.14, P = 0.000, fixed-effect). No severe heterogeneity was checked within the subgroups.

**Table 2:** Overall and subgroup meta-analysis of the association between SNHG20 expression and OS

Subgroup	Studies/N	Patient/N	Pooled HR (95% CI)	P value	Heterogeneity		
					I^2^	P value	Model
Overall	15	1187	2.47 (2.05, 2.98)	0.000	0.00%	0.718	Fixed-effect
Cancer type							
Digestive system cancer	5	483	2.91 (2.16, 3.92)	0.000	0.00%	0.726	Fixed-effect
Osteosarcoma	2	172	1.95 (1.23, 3.09)	0.005	0.00%	0.973	Fixed-effect
Respiratory system cancer	1	42	3.78 (1.18, 12.09)	0.025	–	–	–
Reproductive system cancer	2	153	2.16 (0.95, 4.87)	0.065	0.00%	0.390	Fixed-effect
Head and neck cancer	3	151	1.97 (1.36, 2.85)	0.000	0.00%	0.722	Fixed-effect
Glioma	2	186	3.27 (1.84, 5.82)	0.000	50.00%	0.157	Fixed-effect
Sample size							
>100	4	499	2.86 (2.09, 3.92)	0.000	39.10%	0.177	Fixed-effect
80–100	3	269	2.64 (1.81, 3.87)	0.000	0.00%	0.605	Fixed-effect
<80	8	419	2.09 (1.56, 2.81)	0.000	0.00%	0.929	Fixed-effect
Extracted method							
Direct	5	539	2.55 (1.98, 3.29)	0.000	36.70%	0.176	Fixed-effect
Indirect	10	648	2.38 (1.80, 3.14)	0.000	0.00%	0.903	Fixed-effect

*OS* Overall survival, *HR* hazard ratio, *95% CI* 95% confidence interval

#### SNHG20 expression and DFS/RFS/PFS

**Fig. 3:**
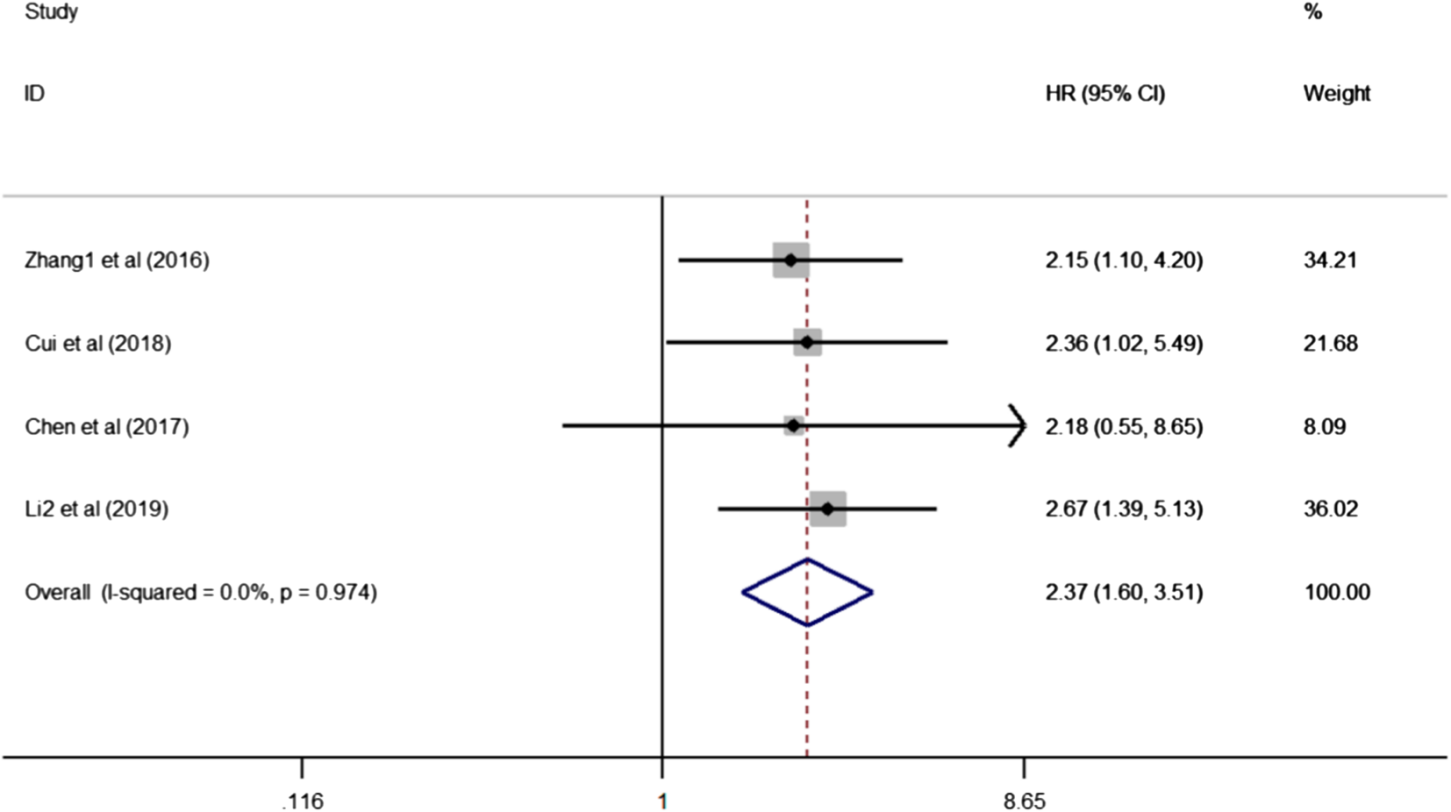
Meta-analysis for the pooled HRs of DFS/RFS/PFS in patients with various cancers. *DFS* disease-free survival, *RFS* recurrence-free survival, *PFS* progression-free survival, *HR* hazard ratio, *95% CI* 95% confidence interval

Four articles consisting of 350 cases exhibited the prognostic role of SNHG20 on cancer progression or recurrence, with a pooled HR of 2.37 (95% CI 1.60–3.51, P = 0.000, Fig. 3). Of note, enforced SNHG20 expression predicted a poor performance for DFS/RFS/PFS in the involved cancer types compared with low SNHG20 expression. No any significant heterogeneity existed across studies under a fixed-effect model (I^2^ = 0.0%, P = 0.974).

### Correlation between SNHG20 expression and clinical characteristics in patients with cancer

#### TNM stage

**Fig. 4:**
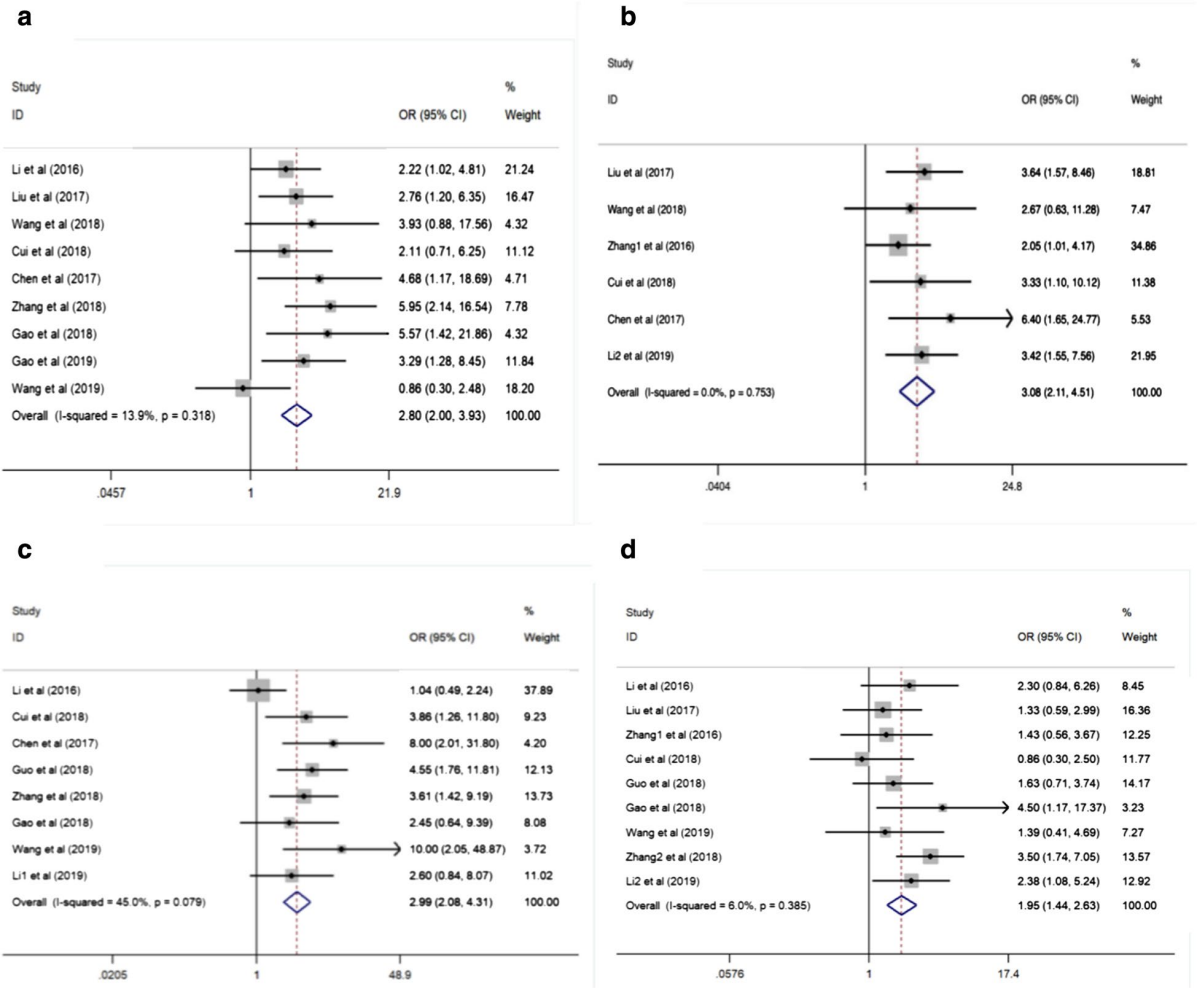
Forests plots for the association between SNHG20 expression and clinicopathological parameters. **a** TNM stage; **b** tumor size; **c** lymph node metastasis; **d** histological grade. *OR* odds ratio, *95% CI* 95% confidence interval

Reports from an aggregate of nine studies declared the correlation of SNHG20 with TNM stage in multiple tumors, with a fixed-effect model on account of limited heterogeneity (I^2^ = 13.9%, P = 0.318). The combined analysis highlighted that patients with elevated SNHG20 expression had a tendency for more advanced TNM phase (OR = 2.80, 95% CI 2.00–3.93, P = 0.000, Fig. 4a, Table 3).

**Table 3:** Meta analysis results for the association of over-expressed SNHG20 with clinicopathological parameters

Categories	Studies (n)	Number of patients	OR (95% CI)	P value	Heterogeneity			Begg	Egger
					I^2^ (%)	P value	Model		
Gender (male vs female)	13	1034	0.96 (0.74, 1.25)	0.763	0.00	0.607	Fixed-effect	0.951	0.792
Smoking status (yes vs no)	3	137	1.16 (0.59, 2.28)	0.676	0.00	0.439	Fixed-effect	–	–
Distant metastasis (yes vs no)	5	502	1.28 (0.35, 4.71)	0.706	84.50	0.000	Random-effect	–	–
TNM stage (III/IV vs I/II)	9	591	2.80 (2.00, 3.93)	0.000	13.90	0.318	Fixed-effect	0.348	0.389
Tumor size (> 5 cm vs < 5 cm)	6	478	3.08 (2.11, 4.51)	0.000	0.00	0.753	Fixed-effect	–	–
Lymph node metastasis (yes vs no)	8	534	2.99 (2.08, 4.31)	0.000	45.00	0.079	Fixed-effect	–	–
Tumor stage (T3/T4 vs T1/T2)	3	203	4.51 (2.17, 9.37)	0.000	0.00	0.943	Fixed-effect	–	–
Histological grade (poorly vs well/moderately)	9	844	1.95 (1.44, 2.63)	0.000	6.00	0.385	Fixed-effect	0.602	0.575

*OR* odd ratio, *95% CI* confidence interval

#### Tumor size

In total, six studies with 478 patients were employed to disclose a link between SNHG20 expression and tumor size. Due to insignificant heterogeneity, a fixed-effect framework was adopted (I^2^ = 0.0%, P = 0.753). Obviously, this association demonstrated that patients with increased SNHG20 expression, were more liable to develop large tumor size (OR = 3.08, 95% CI 2.11–4.51, P = 0.000, Fig. 4b, Table 3).

#### Lymph node metastasis

The relationship between SNHG20 expression and lymph node metastasis was evaluated in eight studies containing 534 patients. A fixed-effect model was applied to calculate the accumulated OR and its 95% CI, when there was marginally moderate heterogeneity between studies (I^2^ = 45.0%, P = 0.079). The aggregated results suggested that patients with up-regulated SNHG20 expression preferentially metastasized to the lymph nodes (OR = 2.99, 95% CI 2.08–4.31, P = 0.000, Fig. 4c, Table 3).

#### Tumor stage

Three studies described the tumor stage of 203 patients in the light of different SNHG20 expression levels. No evidence of statistical heterogeneity was found; consequently, a fixed-effect framework was performed to pool the results (I^2^ = 0.0%, P = 0.943). This showed that the patients with augmented SNHG20 expression tended towards high tumor stage (OR = 4.51, 95% CI 2.17–9.37, P = 0.000, Table 3).

#### Histological grade

There were nine studies revealed a connection between SNHG20 expression and histological grade, and data of 844 patients were collected and pooled for reanalysis. A fixed-effect model was utilized for low heterogeneity detected among included studies (I^2^ = 6.0%, P = 0.385). Statistical analyses illustrated the fact that patients with SNHG20 over-expression had a higher risk of poor histological grade (OR = 1.95, 95% CI 1.44–2.63, P = 0.000, Fig. 4d, Table 3).

Nevertheless, no conspicuous association was observed between SNHG20 expression and gender (OR = 0.96, 95% CI 0.74–1.25, P = 0.763, fixed-effect, Table 3), smoking status (OR = 1.16, 95% CI 0.59–2.28, P = 0.676, fixed-effect, Table 3) or distant metastasis (OR = 1.28, 95% CI 0.35–4.71, P = 0.706, random-effect, Table 3).

### Sensitivity analysis

**Fig. 5:**
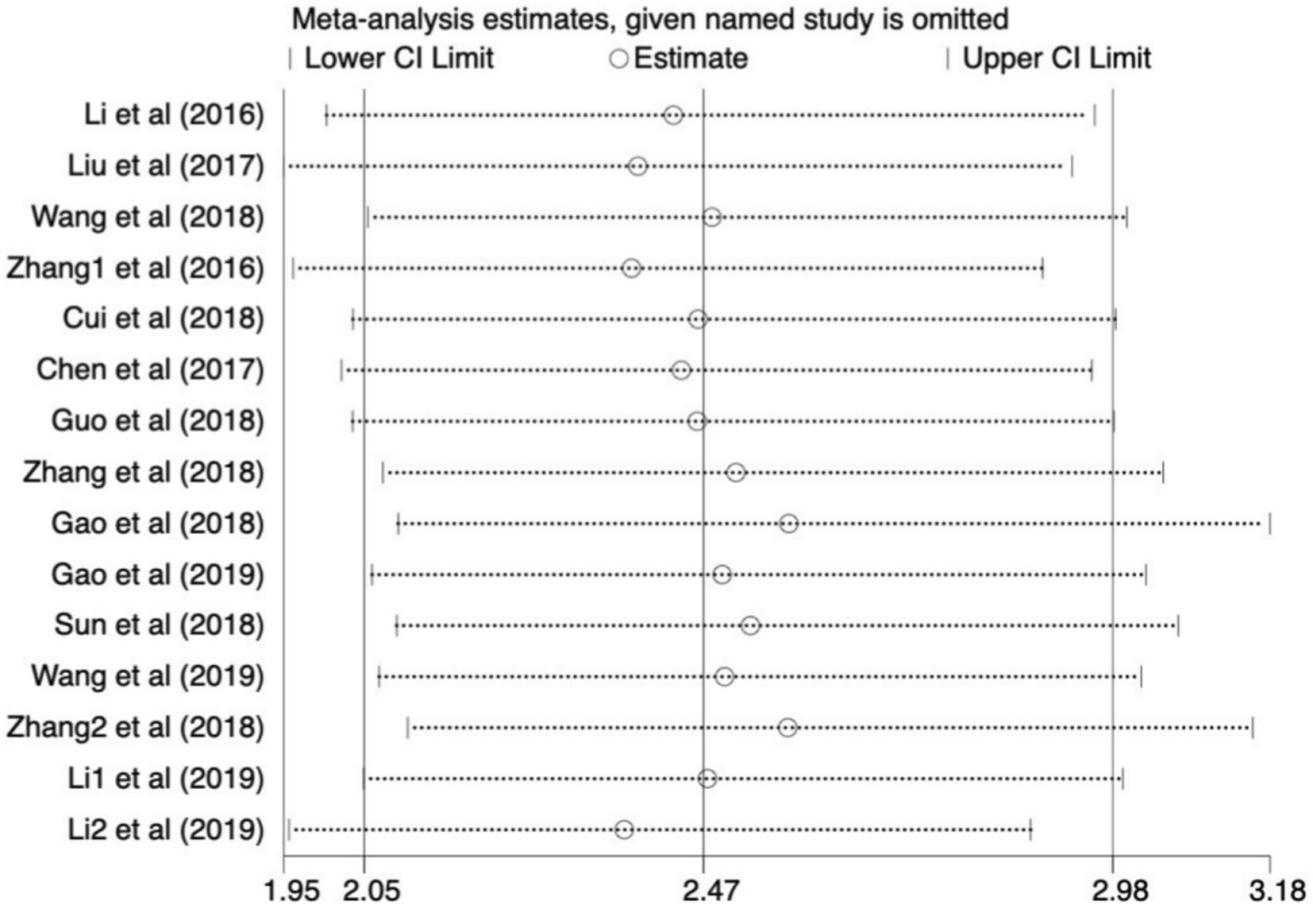
Sensitivity analysis for the correlation between SNHG20 espression with overall survival (OS)

A sensitivity analysis was conducted by omitting individual research by turns with the aim to explore the stability of meta-analysis of SNHG20 and OS. As presented in Fig. 5, the cumulative HR was not dramatically impacted, which further substantiated the reliability and validity of our results.

### Assessment of publication bias

**Fig. 6:**
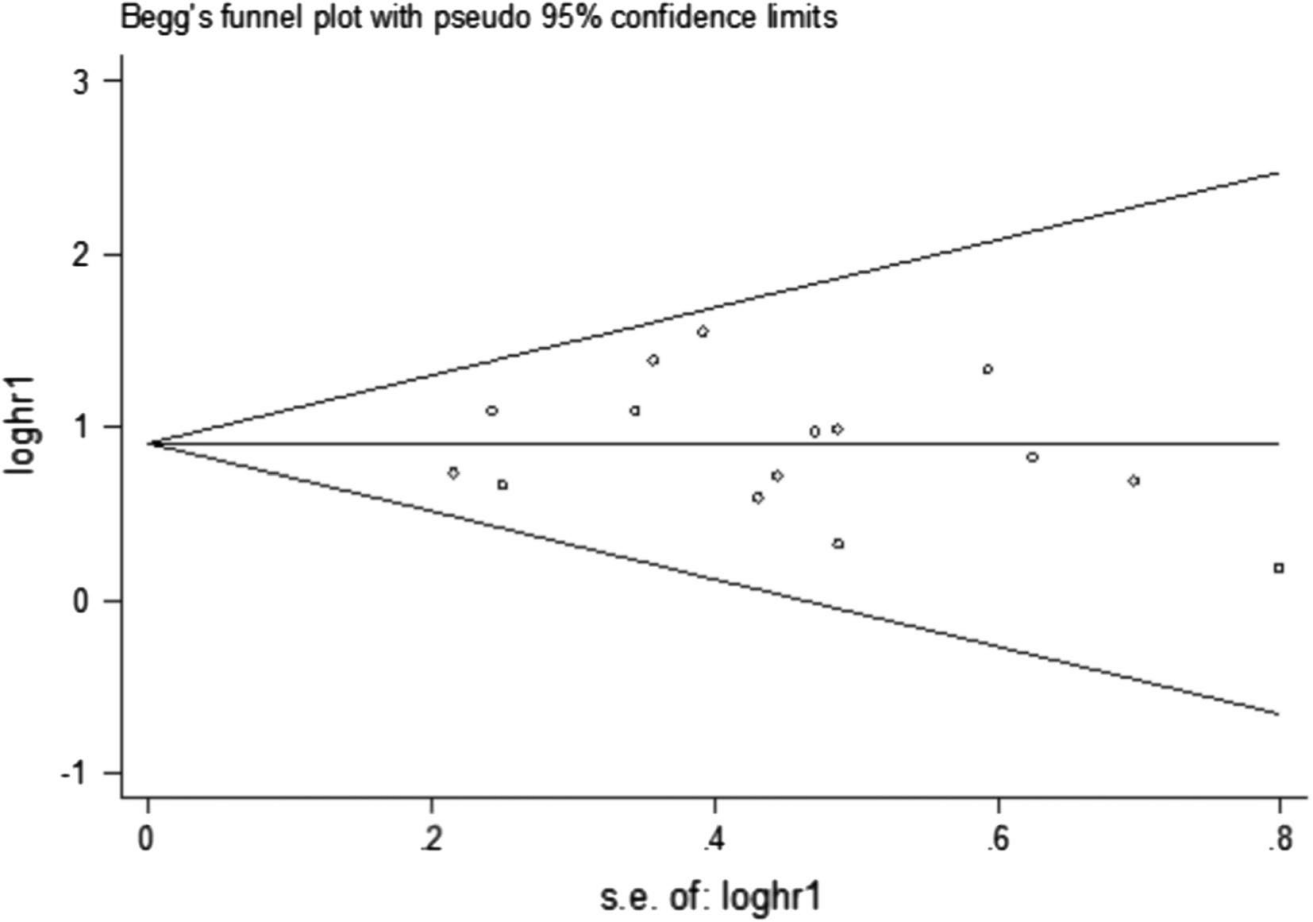
Funnel plot of the publication bias for overall survival (OS)

Publication bias was examined with respect to the survival endpoints of OS by introducing Funnel plots, Begg’s and Egger’s test. The symmetrical funnel plot (Fig. 6), together with the outcomes of Begg’s (P = 0.553) and Egger’s test (P = 0.899), disclosed no distinct publication bias for OS. Furthermore, there was no evidence in favor of publication bias in terms of TNM stage, histological grade, and gender (Table 3). However, analysis of publication bias was inappropriate for tumor size, lymph node metastasis, tumor stage, smoking status, and distant metastasis, owing to the insufficient number of qualified publications in the meta-analysis.

## Discussion

LncRNAs originally considered as transcriptional noise, have today been demonstrated to be implicated in manifold human malignancies [5]. Moreover, dysregulation of lncRNAs has been correlated with cancer cellular development by interfering with alternative splicing of pre-mRNA, by acting as a regulator in the transcription factor and histone-modifying enzyme, or by affecting the steps of translation and protein folding [26, 27]. Hence, the ectopic expression of lncRNAs could have a potential power for monitoring tumors and serving as a promising predictor of survival [28].

Previous published studies have elucidated that SNHG20 was a cancer-related lncRNA and had an indispensable role in oncogenic activity. For instance, SNHG20 has been demonstrated to promote tumor growth through functioning as a competing endogenous RNA (ceRNA) of miR-154 in non-small cell lung cancer and modulating the expression of ZEB2 and RUNX2 [29]. Additionally, SNHG20 could exert its carcinogenic action in breast cancer, and high level of SNHG20 could facilitate the proliferation, invasion and metastasis of cancer cells via modulating miR‐495/HER2 axis [30]. Meanwhile, SNHG20 also elevated tumor progression by controlling the epithelial-to-mesenchymal transition (EMT) signaling pathway in osteosarcoma or hepatocellular carcinoma [23, 24, 31], activating PI3K/Akt/mTOR pathway in glioblastoma [25], as well as upregulating the expression of transforming growth factor-β1 (TGF-β1) in nasopharyngeal carcinoma [18], etc. Given these above molecular mechanisms of SNHG20 among various carcinomas, it was obvious that SNHG20 was connected with an unfavorable prognosis in cancer patients, which further provided support for clinical utility of SNHG20.

We aimed at exploring the relationship between SNHG20 expression levels and prognosis of human carcinomas in the present comprehensive meta-analysis, which pooled a total of 15 independent studies with 1187 tumor sufferers. The results of the research uncovered that enhanced SNHG20 expression was predominantly interrelated with short OS in cancer patients. Subgroup analyses were exploited to maximize clinical relevance. In the subgroup analysis according to cancer types, augmented SNHG20 expression was significantly related to poor OS in respiratory system cancers, gliomas, digestive system cancers, head and neck neoplasms as well as osteosarcomas, but not in cancers of the reproductive system. The reasons for the above phenomenon may be the difference in the age distribution of patients or the origin of tumor cells. Besides, we discovered that neither sample sizes nor data extraction methods altered the overall results. Subsequently, the outcomes from the sensitivity analysis and publication bias assessment also further verified the representativeness and reliability of our analysis. In addition, we identified that there was a strong link between SNHG20 overexpression and unfavorable DFS/RFS/PFS, meaning that cohorts with elevated SNHG20 expression exhibited a higher risk of tumor relapse or progression. Likewise, the clinicopathologic analyses manifested that patients with the high SNHG20 expression levels had increased occurrence probability of advanced TNM stage, large tumor size, positive lymph node metastasis, high tumor stage, and poorly differentiated grade. However, no prominent correlation was found between SNHG20 expression and gender, smoking status or distant metastasis. Taken together, the anomalous modulation of SNHG20 across different kinds of cancers suggests that SNHG20 is qualified as a candidate biomarker for both forecasting poor prognosis and providing therapeutic targets in cancer patients.

Nonetheless, we acknowledge several limitations in this work that should be pointed out. First of all, each and every enrolled study was performed in China, which increases the risk of geographical bias. Second, the sample sizes of some research and the included cancer types were comparatively smaller, which may bring about small-study effects. Third, the Cut-off level of high and low SNHG20 expression level was distinct across studies and not all of them provided this parameter, which perhaps reduces the reliability of our results. Fourth, HR with 95% CI was indirectly reckoned by survival curves in some papers, which are less precise than those directly extracted from the original articles. Last, despite our primary outcomes were lack of heterogeneity, the predictive significance of SNHG20 in multiple human tumors might be exaggerated to some extent. Consequently, high-quality studies that are at large-scale are necessary for the verification of our conclusion.

## Conclusions

In aggregate, the present meta-analysis elucidated that elevated SNHG20 expression is frequent in plenty of various types of cancers and qualified as a dependable and novel predictive factor of poor prognosis and clinicopathological features in cancer patients. Nevertheless, higher-quality multicenter studies with a larger sample capacity are still needed to corroborate and enhance the clinical application of SNHG20 in human malignancies.

## Data Availability

The datasets during the current study are available from the corresponding author on reasonable request.

## Abbreviations

SNHG20: Small nucleolar RNA host gene 20
LncRNAs: Long non-coding RNAs
ceRNA: Competing endogenous RNA
OS: Overall survival
DFS: Disease-free survival
RFS: Recurrence-free survival
PFS: Progression-free survival
qRT-PCR: Quantitative reverse transcription polymerase chain reaction
HCC: Hepatocellular carcinoma
ISH: In situ hybridization
TNM: Tumour‐node‐metastasis
TGF-β1: Transforming growth factor-β1
NOS: Newcastle–Ottawa Scale
EMT: Epithelial-to-mesenchymal transition
HR: Hazard ratio
OR: Odds ratio
CI: Confidence interval
